# The effects of antibiotic exposures on the gut resistome during hematopoietic cell transplantation in children

**DOI:** 10.1080/19490976.2024.2333748

**Published:** 2024-03-30

**Authors:** Sarah M. Heston, Rebecca R. Young, Kirsten Jenkins, Paul L. Martin, Andre Stokhuyzen, Doyle V. Ward, Shakti K. Bhattarai, Vanni Bucci, Mehreen Arshad, Nelson J. Chao, Patrick C. Seed, Matthew S. Kelly

**Affiliations:** a Division of Pediatric Infectious Diseases, Duke University School of Medicine, Durham, NC, USA; b Duke Clinical Research Insitute, Duke University School of Medicine, Durham, NC, USA; c Division of Pediatric Transplant and Cellular Therapy, Duke University School of Medicine, Durham, NC, USA; d Center for Microbiome Research, University of Massachusetts Medical School, Worcester, MA, USA; e Department of Microbiology and Physiological Systems, University of Massachusetts Medical School, Worcester, MA, USA; f Division of Pediatric Infectious Diseases, Ann & Robert H. Lurie Children’s Hospital of Chicago, Chicago, IL, USA; g Division of Hematologic Malignancies and Cellular Therapy, Duke University School of Medicine, Durham, NC, USA

**Keywords:** Shotgun metagenomic sequencing, antibiotic resistance, antimicrobial stewardship, anaerobic bacteria, piperacillin-tazobactam, metronidazole, carbapenem

## Abstract

Antibiotic resistance is a global threat driven primarily by antibiotic use. We evaluated the effects of antibiotic exposures on the gut microbiomes and resistomes of children at high risk of colonization by antibiotic-resistant bacteria. We performed shotgun metagenomic sequencing of 691 serially collected fecal samples from 80 children (<18 years) undergoing hematopoietic cell transplantation. We evaluated the effects of aerobic (cefepime, vancomycin, fluoroquinolones, aminoglycosides, macrolides, and trimethoprim-sulfamethoxazole) and anaerobic (piperacillin-tazobactam, carbapenems, metronidazole, and clindamycin) antibiotic exposures on the diversity and composition of the gut microbiome and resistome. We identified 372 unique antibiotic resistance genes (ARGs); the most frequent ARGs identified encode resistance to tetracyclines (*n* = 88), beta-lactams (*n* = 84), and fluoroquinolones (*n* = 79). Both aerobic and anaerobic antibiotic exposures were associated with a decrease in the number of bacterial species (aerobic, β = 0.71, 95% CI: 0.64, 0.79; anaerobic, β = 0.66, 95% CI: 0.53, 0.82) and the number of unique ARGs (aerobic, β = 0.81, 95% CI: 0.74, 0.90; anaerobic, β = 0.73, 95% CI: 0.61, 0.88) within the gut metagenome. However, only antibiotic regimens that included anaerobic activity were associated with an increase in acquisition of new ARGs (anaerobic, β = 1.50; 95% CI: 1.12, 2.01) and an increase in the relative abundance of ARGs in the gut resistome (anaerobic, β = 1.62; 95% CI: 1.15, 2.27). Specific antibiotic exposures were associated with distinct changes in the number and abundance of ARGs for individual antibiotic classes. Our findings detail the impact of antibiotics on the gut microbiome and resistome and demonstrate that anaerobic antibiotics are particularly likely to promote acquisition and expansion of antibiotic-resistant bacteria.

## Introduction

Antibiotic resistance is one of the most serious global public health threats. In the United States alone, more than 2.8 million antibiotic-resistant infections occur each year, resulting in an estimated $4.6 billion in healthcare expenditures and more than 35,000 deaths.^1,2^ Despite the World Health Organization’s Global Action Plan to combat antibiotic resistance, the number of infections and deaths from antibiotic-resistant organisms continues to rise.^3^ For instance, the number of infections caused by extended-spectrum beta lactamase (ESBL)-producing Enterobacteriaceae has increased by approximately 50% in the United States since 2013.^1^ A number of factors contribute to antibiotic resistance, but use of antibiotics is the major driving force, thereby making hospitals a key setting for antibiotic stewardship efforts and other interventions aiming to reduce the spread of antibiotic-resistant organisms.^1,4^

During hematopoietic cell transplantation (HCT), the recipient’s stem cells are destroyed, leaving the patient profoundly immunosuppressed until the donor stem cells engraft and proliferate. Patients undergoing HCT are at high risk of acquiring antibiotic-resistant organisms due to their prolonged hospitalizations, impaired immunity, and frequent receipt of broad-spectrum antibiotics. Colonization by multidrug-resistant bacteria is observed in more than 50% of adult HCT recipients and has been associated with a higher incidence of non-relapse-related mortality.^5^ Moreover, HCT recipients have a high incidence of antibiotic-resistant infections arising from the gut because of gut dysbiosis, impaired host immunity, and mucosal barrier injury from chemotherapy or radiation therapy.^6,7^ Overgrowth of potential pathogens that colonize the gut was shown in several studies to precede bloodstream infections caused by antibiotic-resistant organisms.^7,8^ In addition, antibiotics with an anaerobic spectrum of activity are increasingly recognized to disrupt the gut microbiome and are associated with poor outcomes after HCT including a higher risk of graft-versus-host disease (GVHD).^9^ Historically, studies of HCT recipients identified colonization by antibiotic-resistant bacteria using culture- or PCR-based methods. These approaches are limited because they enable study only of cultivable bacteria or a small number of specific antibiotic resistance genes (ARGs). With the development of next-generation sequencing methods, we are now able to simultaneously detect the full complement of ARGs within a sample, collectively referred to as the “resistome.”

In this study, we used shotgun metagenomic sequencing of 691 fecal samples from 80 children and adolescents undergoing HCT. We evaluated the impact of antibiotics with aerobic and anaerobic spectra of activity on gut microbiome composition and the gut resistome. In addition, we evaluated associations between antibiotic exposures and the number and abundances of ARGs to specific antibiotic classes to describe the effects of specific antibiotics on the gut resistome.

## Results

### Patient and sample characteristics

We longitudinally collected fecal samples from children (<18 years) undergoing HCT at Duke University Hospital between 2015 and 2018; sample collection started prior to HCT and continued until 100 days after HCT. Our cohort included 80 children with a median (interquartile range [IQR]) age of 5.1 (2.2, 12.8) years (Table 1). The most frequent indication for HCT was hematologic malignancy (41%), and most transplants were allogeneic (88%), with umbilical cord blood being the most common donor source (64%). Cefepime, vancomycin, and trimethoprim-sulfamethoxazole (TMP-SMX) were the most commonly received antibiotics (Table 2). Fifty-eight (73%) subjects received one or more antibiotics with an anaerobic spectrum of activity, including 44 (55%) subjects who received metronidazole, 26 (33%) who received piperacillin-tazobactam, 15 (19%) who received a carbapenem, and four (5%) who received clindamycin. After quality filtering and sample pruning, 691 fecal samples [median (IQR) of 8 (6, 12) samples per child] were included in downstream analyses. Shotgun metagenomic sequencing of these samples generated a total of 5,516,212,036 high-quality, paired-end metagenomic reads with a median (IQR) sequencing depth of 7.23 (3.20, 11.96) million reads per sample.

**Table 1.** Characteristics of the study population of 80 children and adolescents undergoing HCT.

			N	%
Median (IQR) age, years			5.1	(2.2, 12.8)
Female sex			34	43
HCT indication				
	Hematologic malignancy		33	41
	Immunodeficiency		7	9
	Non-malignant hematologic disorder		14	18
	Metabolic disease		18	23
	Solid tumor		8	10
HCT type				
	Allogeneic		70	88
		Bone marrow	18	26
		Umbilical cord blood	51	73
		Other	1	1
	Autologous		10	13
Conditioning regimen				
	Myeloablative		77	96
	Non-myeloablative/RIC		3	4
Median (IQR) day of engraftment			19	(15, 22)
Acute GVHD of gut or liver			23	29
Bloodstream infection			22	28
Two-year all-cause mortality			20	25

IQR, interquartile range; HCT, hematopoietic cell transplantation; RIC, reduced-intensity conditioning; GVHD, graft-versus-host disease.

**Table 2.** Prevalence of antibiotic exposures among the study population and fecal samples collected after antibiotic exposures.

	Subject (*N* = 80)		Samples (*N* = 691)	
Antibiotic	N	%	N	%
Cefepime	72	90	258	37
Vancomycin	73	91	175	25
TMP-SMX	71	89	162	23
Metronidazole	44	55	98	14
Piperacillin-tazobactam	26	33	65	9
Macrolide	19	24	36	5
Other beta-lactam	15	19	58	8
Carbapenem	15	19	27	4
Aminoglycoside	10	13	13	2
Fluoroquinolone	8	10	32	5
Clindamycin	4	5	11	2
Tetracycline	1	1	3	0

TMP-SMX, trimethoprim-sulfamethoxazole.

### Composition of the gut microbiome during HCT

**Figure 1.**
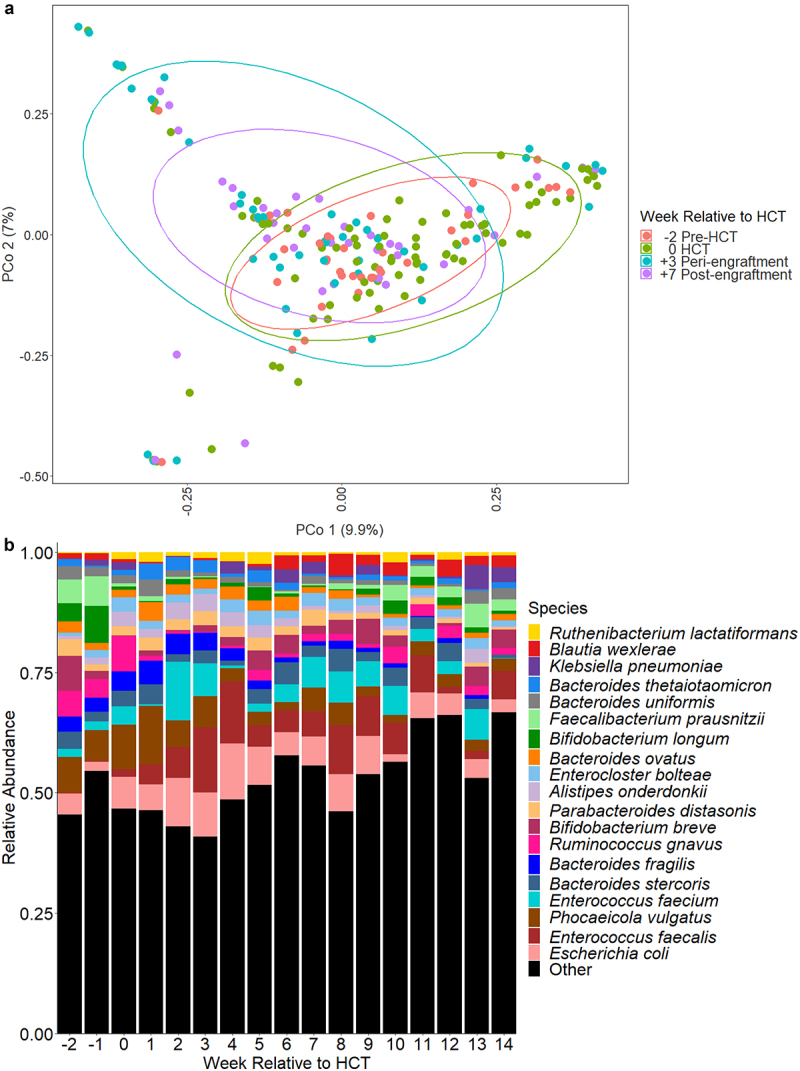
Microbiome composition over time relative to HCT. a. Principle coordinate analysis of Bray-Curtis distances for microbiome composition at weeks − 2, 0, +3, and + 7. These timepoints correlate to clinically-relevant periods relative to HCT, specifically pre-transplant, time of HCT, peri-engraftment, and post-engraftment. Each dot represents a single sample; the circles represent clustering of samples by timepoint. b. Relative abundances of highly abundant bacterial species in fecal samples of children by week relative to hematopoietic cell transplantation (HCT). *Escherichia coli*, *Enterococcus faecalis*, and *phocaeicola vulgatus* (formerly *bacteroides vulgatus*) were the most abundant bacterial species. The relative abundances of several anaerobic species (e.g., *Bifidobacterium longum, faecalibacterium prausnitzii, parabacteroides distasonis*) declined over time, whereas the relative abundances of potential pathogens (e.g., *E. faecalis*, *Klebsiella pneumoniae*) increased after HCT.

We identified a total of 1095 bacterial species with a median (IQR) of 36 (17, 67) unique bacterial species per sample. The most highly abundant bacterial species across all samples were *Escherichia coli* (mean relative abundance of 6.4%), *Enterococcus faecalis* (5.6%), *Phocaeicola vulgatus* (formerly *Bacteroides vulgatus*, 5.4%), *Enterococcus faecium* (4.1%), and *Bacteroides stercoris* (2.8%). The composition of the gut microbiome underwent substantial changes during the course of HCT (Figure 1); specifically, the relative abundances of several anaerobic species (e.g., *Bifidobacterium longum, Faecalibacterium prausnitzii, Parabacteroides distasonis, Phocaeicola vulgatus*, and several *Bacteroides* species) declined, and the relative abundances of potential pathogens (e.g., *E. faecalis*, *Klebsiella pneumoniae*, and viridans group streptococci) increased during the course of HCT (Supplementary Table S1). The number of new bacterial species acquired since the previously sequenced sample increased by 1% (negative binomial regression; 95% confidence interval [CI]: 0.8%, 1.2%, *p* < .0001) per day after HCT (Supplementary Table S2), whereas there were no significant changes in the total number of bacterial species or the fluctuation of the gut microbiome as measured by the Jaccard distance with increasing time since HCT. This suggests that despite the substantial compositional shifts during HCT, the gut microbiome becomes colonized by new bacterial species at a similar rate as it loses bacterial species during the transplant course.

Exposure to aerobic antibiotics was associated with a 29% (negative binomial regression; 95% CI: 21%, 36%, *p* < .0001) decrease in the number of species and a 0.1 (linear regression; 95% CI: 0.05, 0.14, *p* < .0001) increase in gut microbiome fluctuation as measured by the Jaccard distance. Anaerobic antibiotic exposures were similarly associated with a 34% (negative binomial regression; 95% CI: 18%, 47%, *p* = .0001) decrease in the number of species and a 0.15 (linear regression; 95% CI: 0.07, 0.22, *p* = .0001) increase in microbiome fluctuation. Interestingly, exposure to both aerobic and anaerobic antibiotics was associated with a 54% (negative binomial regression; 95% CI: 46%, 60%, *p* < .0001) decrease in the number of species and a 0.17 (linear regression; 95% CI: 0.12, 0.22, *p* < .0001) increase in microbiome fluctuation (Supplementary Table S2). Finally, while aerobic antibiotic exposure had no effect on the acquisition of new bacterial species, there was a 45% (negative binomial regression; 95% CI: 10%, 90%, *p* = .008) increase in the number of acquired species associated with anaerobic antibiotic exposures (Supplementary Table S2). Taken together, both aerobic and anaerobic antibiotic exposures were associated with losses of bacterial species from the gut microbiome and an increase in fluctuation of gut microbiome composition, an effect that seemed to be exaggerated by concomitant exposure to aerobic and anaerobic antibiotics. However, only anaerobic antibiotic exposures were associated with an increase in colonization of the gut by new bacterial species.

**Figure 2.**
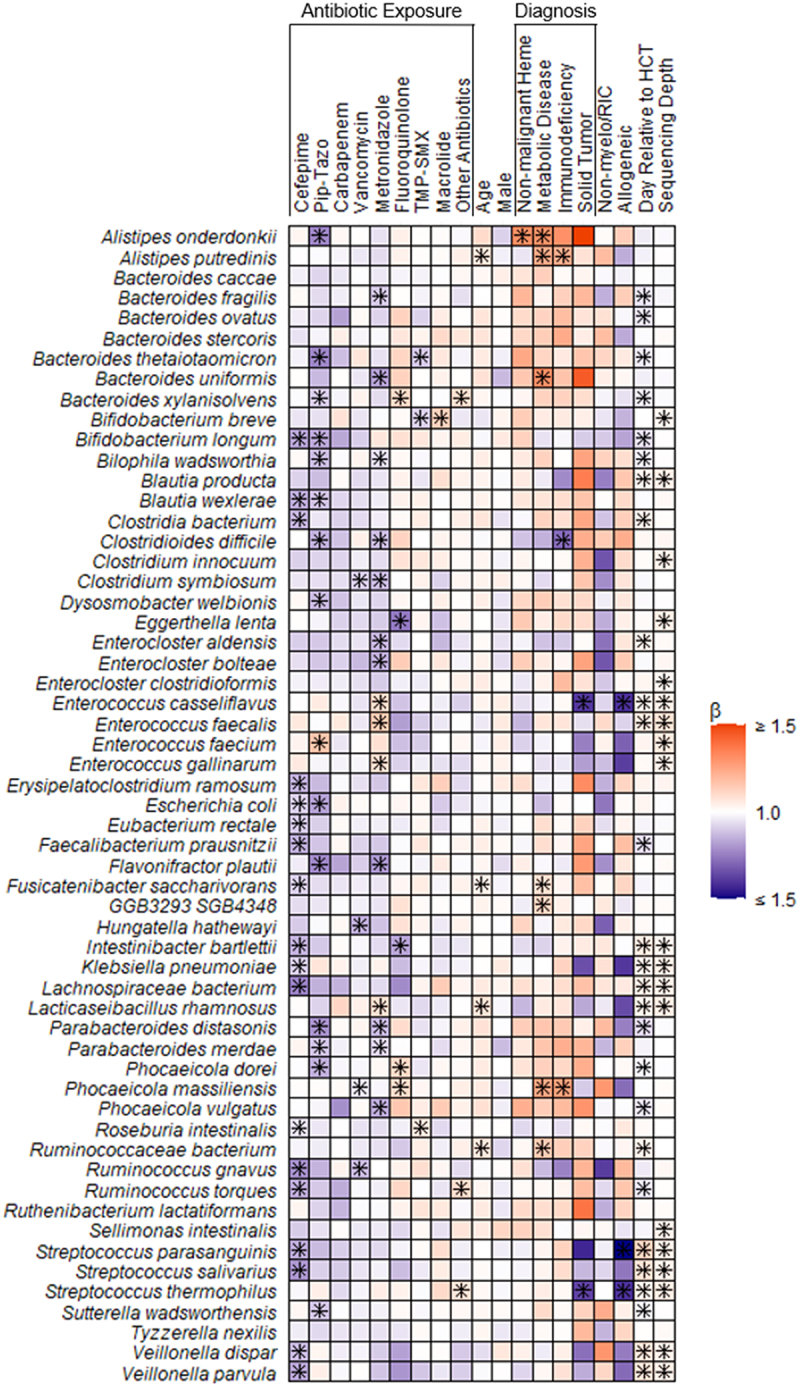
Heatmap depicting associations between antibiotic exposures and other clinical factors and gut microbiome composition. Associations were estimated from linear mixed effects models fit using MaAsLin2. We included bacterial species with a minimum mean relative abundance of 1% and a sample prevalence of 5%; the false discovery rate was set to 0.1. Broadly, there were declines in the relative abundances of several bacterial species associated with antibiotic exposures. Notably, metronidazole and piperacillin-tazobactam exposures were associated with increases in the relative abundances of several enterococcal species. “Other” antibiotics includes exposures to tetracyclines, clindamycin, aminoglycosides, and non-cefepime beta-lactams. Betas indicate effect sizes and asterisks denote significant changes. Pip-tazo, piperacillin-tazobactam; TMP-SMX, trimethoprim-sulfamethoxazole; non-malignant heme, non-malignant hematologic disorder; non-myelo/RIC, non-myeloablative and reduced-intensity conditioning.

We next sought to evaluate associations between exposures to specific antibiotics and changes in the composition of the gut microbiome (Figure 2). Cefepime exposure was associated with decreases in the relative abundances of both anaerobic bacteria from the genera *Bifidobacterium, Blautia*, and *Clostridium*, as well as potential pathogens including *E. coli*, *K. pneumoniae*, and viridans group streptococci. The anaerobic antibiotics piperacillin-tazobactam and metronidazole were associated with decreases in the relative abundances of several anaerobic bacterial species, including *Bacteroides thetaiotaomicron, Bacteroides uniformis, B. longum, Flavonifractor plautii, Parabacteroides distasonis*, and *P. vulgatus*. Piperacillin-tazobactam and metronidazole were also notably associated with increases in the relative abundances of *E. faecalis, E. faecium*, and *Enterococcus gallinarum*. Increasing enterococcal colonization was notable, as this genus was previously shown to be a risk factor for GVHD-related mortality and antibiotic-resistant bloodstream infections among adult HCT recipients.^8,10^

### The gut resistome during HCT

We detected a total of 372 unique ARGs with a median (IQR) of 29 (16, 57) ARGs per sample. The most common ARGs detected were tet32, tetW, tetO, and dfrF, each of which were present in more than 60% of samples and can confer resistance to tetracyclines and trimethoprim, respectively. The antibiotic classes with the most identified ARGs were tetracyclines, beta-lactams, and fluoroquinolones (Table 3). Compared to individuals who did not experience these outcomes, there were no substantial differences in ARG relative abundances over time among participants who developed acute gut or liver GVHD, bloodstream infection, or two-year all-cause mortality (Supplementary Figure S1).

**Table 3.** Antibiotic resistance genes identified in fecal samples that may confer resistance to clinically relevant antibiotics.

Resistance Gene Class	Number of Genes Detected	Median (IQR) Number of ARGs per Sample	Median (IQR) ARG Abundance per Sample (RPKM)
Tetracycline^a^	88	9 (5, 15)	412 (210, 742)
Beta-lactam^b^	84	5 (2, 16)	116 (17, 473)
Fluoroquinolone	79	6 (2, 19)	118 (5, 589)
Macrolide	73	6 (3, 12)	243 (98, 605)
Aminoglycoside	68	4 (1, 8)	112 (19, 412)
Phenicol	56	1 (1, 6)	8 (0, 79)
Carbapenem^c^	41	2 (0, 3)	11 (0, 82)
Lincosamide^d^	31	3 (1, 4)	117 (34, 242)
Diaminopyrimidine^e^	30	2 (1, 3)	40 (8, 131)
Glycopeptide^f^	30	0 (0, 2)	0 (0, 7)
Sulfonamide	8	0 (0, 1)	0 (0, 6)
Oxacolidinone^g^	5	0 (0, 0)	0 (0, 0)
Nitromidazole^h^	3	0 (0, 1)	0 (0, 21)

IQR, interquartile range; RPKM, reads per kilobase million mapped reads.

^a^Tetracycline includes tetracycline and glycylcycline (e.g., tigecycline) resistance genes.

^b^Beta-lactam includes cephalosporin, cephamycin, penam, and monobactam resistance genes.

^c^Carbapenem includes carbapenem and penem resistance genes

^d^incosamide includes clindamycin resistance genes

^e^Diaminopyrimidine includes trimethoprim resistance genes

^f^Glycopeptide includes vancomycin resistance genes

^g^Oxazolidinone includes linezolid resistance genes

^h^Nitromidazole includes metronidazole resistance genes

**Figure 3.**
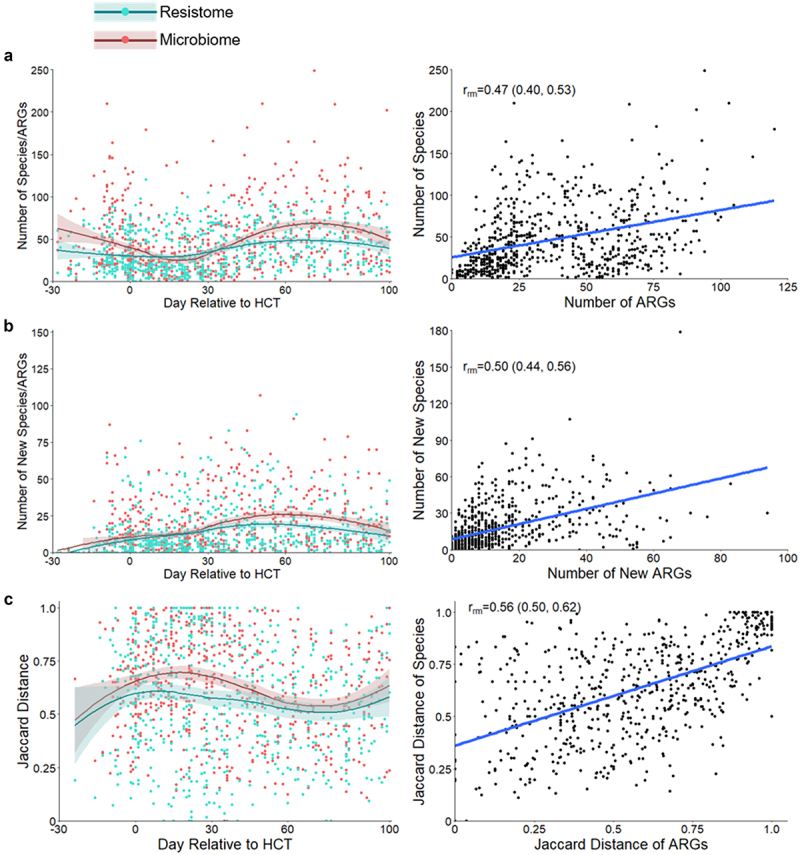
Changes to the gut resistome and microbiome over time. a. Number of ARGs from the gut resistome and number of bacterial species from the gut microbiome in fecal samples by day relative to HCT; repeated measures correlation for the number of bacterial species and number of ARGs per sample. b. Number of new ARGs in the gut resistome and bacterial species in the gut microbiome by day relative to HCT; repeated measures correlation of the number of new bacterial species and ARGs since the previous sample. c. Jaccard distances for the gut resistome and microbiome by day relative to HCT; correlation of the Jaccard distances of the microbiome and resistome. Measures of the resistome and microbiome had similar trends over time and were moderately correlated when accounting for repeated measures. Jaccard distance values closer to one represent increased fluctuation. Points represent individual fecal samples; the smoothed lines were created using the Loess function, and shaded areas represent the 95% confidence intervals. The gut resistome is represented in blue, and the gut microbiome is shown in red. ARG, antibiotic resistance gene; HCT, hematopoietic cell transplantation; r_rm_, repeated measures correlation coefficient.

The gut resistome of children was highly dynamic during the course of HCT. There was a 0.4% (gamma regression; 95% CI: 0.1%, 0.7%, *p* = .009); increase in the overall relative abundance of ARGs within the gut. There was also a 0.2% (negative binomial regression; 95% CI: 0.1%, 0.4%, *p* = .003) increase in the number of ARGs and a 0.8% (negative binomial regression; 95% CI: 0.6%, 1.1%, *p* < .0001) increase in the number of new ARGs acquired since the previously sequenced sample with each day during HCT. The overall fluctuation of the gut resistome decreased over time during HCT (linear regression; β=-0.0008; 95% CI: −0.002, −0.00005, *p* = .04; Figure 3; Supplementary Table S2). Increasing age was associated with a 5% (gamma regression; 95% CI: 1%, 8%, *p* = .01) decrease in gut resistome abundance and a decrease in resistome fluctuation (linear regression; β=-0.008; 95% CI: −0.01, −0.001, *p* = .03); other subject and transplant factors were not associated with measures of the resistome. Thus, accounting for age and other clinical factors, new ARGs are acquired during HCT and the overall stability of the resistome increases with time.

**Figure 4.**
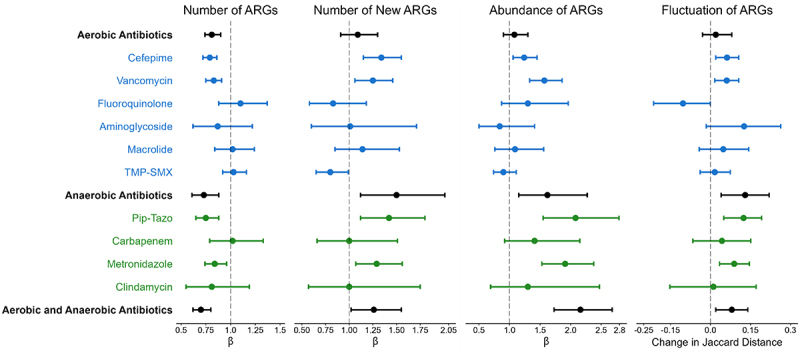
Effect of aerobic and anaerobic antibiotics on measures of the gut resistome. The effect of aerobic and anaerobic antibiotics is shown for the following measures of the gut resistome: the number of ARGs per sample, the number of new ARGs per sample, the abundance of ARGs, and gut resistome fluctuation, as measured by the Jaccard distance. All antibiotic exposures were associated with a decrease in the number of ARGs; however, only antibiotic exposures that included anaerobic antibiotics were associated with increases in the number of new ARGs acquired since the previous sample, the abundance of ARGs, and the fluctuation of the resistome. Points represent estimates, and error bars denote 95% confidence intervals. ARG, antibiotic resistance gene; TMP-SMX, trimethoprim-sulfamethoxazole; Pip-Tazo, piperacillin-tazobactam.

Antibiotic exposures were a major driver of changes in the gut resistome during HCT. Compared to no antibiotic exposure, aerobic antibiotics were associated with a 19% (negative binomial regression; 95% CI: 10%, 26%, *p* < .0001) decrease in the number of ARGs, but not with a change in the number of new ARGs, the abundance of ARGs, or fluctuation of the gut resistome. These findings suggest that aerobic antibiotic exposure is associated with a loss of ARG richness but also with an accompanying expansion of bacteria harboring the remaining ARGs (Supplementary Table S2; Figure 4). Conversely, anaerobic antibiotic exposures were associated with a 27% (negative binomial regression; 95% CI: 12%, 39%, *p* = .0008) decrease in the number of ARGs, a 50% (negative binomial regression; 95% CI: 12%, 101%, *p* = .006) increase in acquisition of new ARGs, a 62% (gamma regression; 95% CI: 15%, 127%, *p* = .005) increase in the abundance of ARGs, and a 0.13 (linear regression; 95% CI: 0.04, 0.22, *p* = .003) increase in the fluctuation of the gut resistome compared to no anaerobic antibiotic exposure (Figure 4). Exposures to both aerobic and anaerobic antibiotics were associated with similar effects (Supplementary Table S2). When considered individually, there were antibiotic-specific effects on measures of the gut resistome (Figure 4; Supplementary Table S3). Thus, while both aerobic and anaerobic antibiotics led to a decrease in the number of ARGs, only antibiotic regimens that included anaerobic antibiotic exposures were associated with acquisition of new ARGs and with expansion of the abundance of the gut resistome.

We next evaluated correlations between measures of gut microbiome composition and the gut resistome using repeated measures correlation. There were moderate correlations between the number of ARGs and bacterial species (repeated measures correlation; r_rm_ = 0.47; 95% CI: 0.40, 0.53, *p* < .0001), the number of newly acquired ARGs and bacterial species (r_rm_ = 0.50; 95% CI: 0.44, 0.56, *p* < .0001), and the stability of the gut microbiome and resistome (r_rm_ = 0.56; 95% CI: 0.50, 0.62, *p* < .0001; Figure 3). These data suggest that gut microbiome richness is a major factor in determining the extent of the gut resistome, and antibiotic-related losses of gut microbial richness are generally associated with a reduction in the richness of the gut resistome.

### Antibiotic exposures affect same and different ARG classes

**Figure 5.**
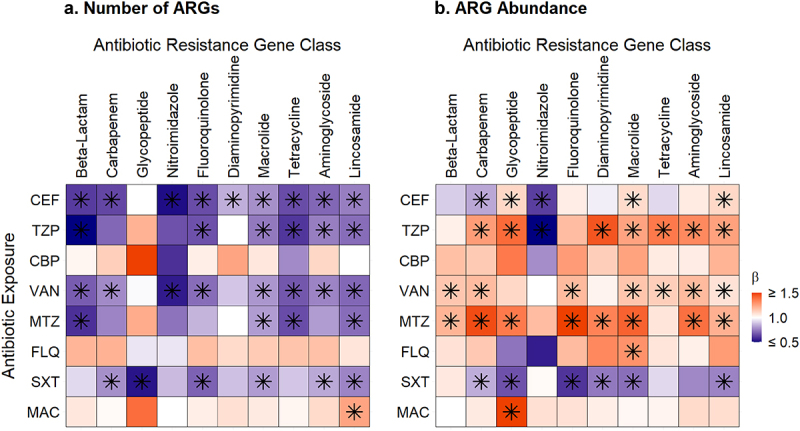
Heatmaps of associations between antibiotic exposures and ARGs conferring resistance to specific antibiotic classes. a. Antibiotic exposure effect on the number of ARGs for the exposure antibiotic class and others. b. Antibiotic exposure effect on the ARG abundance for the exposure antibiotic class and others. Broadly, exposure to an antibiotic of one class was associated with decreases in the number of ARGs and increases in the relative abundances of ARGs that confer resistance to several other classes of antibiotics. Beta values correspond to effect sizes from mixed effect models with individual antibiotic exposures as fixed effects and subject as a random effect. ARGs, antibiotic resistance genes, FEP, cefepime; TZP, piperacillin-tazobactam; CBP, carbapenem; VAN, vancomycin; MTZ, metronidazole; FLQ, fluoroquinolone; SXT, trimethoprim-sulfamethoxazole; MAC, macrolide. Tetracycline includes tetracycline and glycylcycline ARGs; beta-lactam includes cephalosporin, cephamycin, penam, and monobactam ARGs; lincosamide ARGs confer resistance to clindamycin; carbapenem includes carbapenem and penem ARGs; glycopeptide ARGs confers resistance to vancomycin; diaminopyrimidine ARGs confer resistance to trimethoprim; nitromidazole ARGs confer resistance to metronidazole. Asterisks denote significant change.

Finally, we determined the effect of specific antibiotic exposures on the number and abundance of ARGs of the same antibiotic class and other antibiotic classes (Figure 5; Supplementary Tables S4 and S5). Cefepime was associated with decreases in the number and abundance of ARGs for multiple ARG classes, including ARGs that confer resistance to beta-lactams (negative binomial regression, number β = 0.66, 95% CI: 0.57, 0.76, *p* < .0001) and carbapenems (negative binomial regression, number β = 0.68, 95% CI: 0.58, 0.80, *p* < .0001; gamma regression, abundance β = 0.83, 95% CI: 0.83, 0.84, *p* < .0001). Piperacillin-tazobactam and metronidazole were associated with decreases in the number of ARGs from many antibiotic classes but with increases in the relative abundances of some classes of ARGs, especially glycopeptide ARGs (gamma regression, abundance, piperacillin-tazobactam β = 2.22, 95% CI: 1.29, 3.83, *p* = .008; metronidazole β = 2.10, 95% CI: 1.44, 3.07, *p* = .0003) that confer resistance to vancomycin. Vancomycin exposure was associated with significant decreases in the number of ARGs but increases in the relative abundance of ARGs that confer resistance to several classes of antibiotics. Notably, vancomycin exposure was associated with a 28% (95% CI: 16%, 38%, *p* = .0001) decrease in the number of ARGs and a 48% (95% CI: 46%, 49%, *p* < .0001) increase in the relative abundance of ARGs that confer resistance to beta-lactams; a 25% (95% CI: 12%, 36%, *p* = .0008) decrease in the number and a 58% (95% CI: 16%, 115%, *p* = 0.006) increase in the relative abundance of ARGs that confer resistance to fluoroquinolones; and a 21% (95% CI: 9%, 31%, *p* = .001) decrease in the number and a 58% (95% CI: 57%, 58%, *p* < .0001) increase in the relative abundance of ARGs that confer resistance to aminoglycosides. Finally, though the associations did not reach statistical significance, fluoroquinolone exposure tended to be associated with an increased number of ARGs and an increased relative abundance of ARGs that confer resistance to several classes of clinically-relevant antibiotics, including beta-lactams, carbapenems, fluoroquinolones, and aminoglycosides.

## Discussion

Using shotgun metagenomics, we demonstrated the dynamic changes in the gut microbiome and resistome that occur among children undergoing HCT. We described the effects of antibiotics on both the composition of the gut microbiome and resistome, including the distinct effects of aerobic and anaerobic antibiotics on gut microbiome composition and on the extent of the gut resistome.

Compared to previous culture- and PCR-based methodologies, use of shotgun metagenomics is a novel way to study the complex, dynamic reservoir of ARGs within the gut.^11^ Considering ARGs collectively is particularly important because commensal bacteria, including those from the phyla Bacteroidetes and Firmicutes, likely harbor most of the ARGs in the healthy human gut and serve as a potential source of horizontal transfer of ARGs to common enteric pathogens.^11,12^ Colonization by commensal, anaerobic bacteria with ARGs may also explain how ARGs are detected even in the absence of antibiotic pressure.^13^ In keeping with this observation, we detected a preponderance of ARGs that confer resistance to tetracyclines, macrolides, fluoroquinolones, and aminoglycosides despite the study population being infrequently exposed to these antibiotic classes. Knowledge of gut colonization by any bacteria with ARGs is especially important in the HCT population, as these patients are at high-risk of antibiotic-resistant bloodstream infections caused by bacteria arising from the gut.

Surprisingly little is known about the dynamics of the gut resistome following HCT. A small cohort study of eight children undergoing HCT for high-risk acute leukemia found that the most commonly detected ARGs conferred resistance to tetracyclines, macrolides, beta-lactams, and aminoglycosides, as was observed in our cohort.^14^ The authors of this study also concluded that the four children who developed acute GVHD had a gut resistome signature that was distinct from that of children who did not develop acute GVHD, with acquisition of several new ARGs after HCT.^14^ Though somewhat beyond the scope of the current analysis, we generally did not observe a substantial difference in the relative abundances of ARGs among children who developed acute GVHD of the gut or liver. Additionally, the study by D’Amico et al. was limited by its small sample size and did not assess the effect of antibiotics on the gut resistome.^14^ Our findings suggest that antibiotics significantly impact both gut microbiome composition and the gut resistome during HCT. A recent study evaluated antibiotic exposures on the gut resistome of adult HCT recipients.^15^ However, due to sampling limitations, the authors conducted less powerful statistical analyses as those presented here.^15^ They were unable to account for clinical factors that affect the gut microbiota, including intrapersonal differences, which are a substantial source of variation in microbiome-related analyses.^15,16^ Due to the correlations between the microbiome and resistome and the similar loss of ARGs and species associated with antibiotic exposures in our study, we concluded that changes to the resistome are largely impacted by changes in the microbiome composition. Specifically, antibiotic exposures lead to loss of bacterial species from the gut microbiome and this loss of species results in contraction of the number of ARGs in the gut resistome. This finding could be considered contrary to traditional principles of antimicrobial resistance, wherein an antibiotic exposure serves as isolated selective pressure for antibiotic-resistant pathogens.^17^ However, our findings confirm that antibiotic exposures have much broader impacts on both pathogenic and health-promoting bacteria and the collection of antibiotic resistance genes harbored by the gut microbiota.

We found that aerobic and anaerobic antibiotics had distinct effects on gut microbiome composition and the gut resistome. These findings are notable because anaerobic bacteria in the gut are primarily responsible for colonization resistance – the ability to prevent colonization by exogenous bacteria – through a variety of interspecies and microbe-host interaction mechanisms.^18^ Thus, loss of anaerobic bacteria with anaerobic antibiotic exposures likely reduces colonization resistance, leading to expansion of existing antibiotic-resistant bacteria or the acquisition of exogenous antibiotic-resistant bacteria.^19^ Loss of colonization resistance with anaerobic antibiotics likely explains our findings of increased ARG acquisition, ARG abundance, and resistome fluctuation despite the overall loss of ARGs associated with anaerobic antibiotics. Piperacillin-tazobactam and metronidazole were associated with lower relative abundances of *Bacteroides, Bifidobacterium, Blautia*, and *Clostridium* species, higher relative abundances of *Enterococcus* species, and an increased abundance of glycopeptide ARGs commonly found in enterococci. While we cannot directly assign ARGs to specific bacteria, these associations support the conclusion that anaerobic antibiotic exposures result in loss of colonization resistance from anaerobes and colonization or expansion of enterococci within the gut microbiome. Taken together, our findings provide further evidence that anaerobic antibiotics should be used judiciously in this high-risk population.^9^

Our data also suggest that exposure to an antibiotic of one class can lead to acquisition of collateral antibiotic resistance to other antibiotic classes. In our cohort, vancomycin, piperacillin-tazobactam, and metronidazole exposures were associated with increases in the relative abundances of several other ARG classes, including fluoroquinolone, macrolide, tetracycline, and aminoglycoside ARGs. This phenomenon has been demonstrated in healthy adults, among whom ciprofloxacin led to an increase in class D beta-lactamases and macrolide ARGs, and in premature neonates, in whom meropenem led to higher relative abundances of ARGs that confer resistance to fluoroquinolones, macrolides, tetracyclines, and trimethoprim.^20,21^ This expansion of collateral antibiotic resistance has likely also been observed clinically in the HCT population. In a meta-analysis of adults undergoing HCT, there was increased odds of carbapenem-resistant *Klebsiella pneumoniae* infections not only with carbapenem exposure, but also with aminoglycoside, glycopeptide, and quinolone exposures.^22^ Similarly, infection with multidrug-resistant *Pseudomonas aeruginosa* was associated with vancomycin exposure in a meta-analysis of HCT recipients.^23^ Our data suggest that antibiotic pressure selects for organisms that are resistant to the exposure agent and also facilitates acquisition of new bacterial species with ARGs encoding resistance to diverse antibiotic classes.

The effect of fluoroquinolone exposure on collateral antibiotic resistance is of particular interest, given that levofloxacin is the preferred agent for antibacterial prophylaxis after HCT.^24^ Fluoroquinolone use during chemotherapy-induced neutropenia has been associated with a lower risk of febrile neutropenia and bloodstream infections among adults and children with hematologic malignancies, though its effectiveness in preventing these complications among pediatric HCT recipients is less clear. ,^25–27^ Additionally, it has more recently been associated with increased risks of colonization and infection by carbapenem-resistant Enterobacteriaceae.^28^ Moreover, in a murine model, ciprofloxacin exposure led to an increase in the abundance of beta-lactamase ARGs.^29^ We found increases in both the richness and abundance of ARGS for several different antibiotic classes with fluoroquinolone exposure, although fluoroquinolone exposures were relatively infrequent in our cohort and few of these associations were statistically significant. On the contrary, in a study of 49 children with acute lymphoblastic leukemia, levofloxacin prophylaxis was associated with an increase in the prevalence of fluoroquinolone resistance, while a rise in collateral antibiotic resistance from other ARG classes was not observed.^30^ As levofloxacin prophylaxis is becoming more common in pediatric centers, further research is needed to determine the effect of levofloxacin on the gut resistome.

Our study has several notable limitations. First, our study definition of antibiotic exposure is likely underestimating the effect of antibiotics on acquisition of ARGs, given that antibiotic-induced changes in the gut microbiome are known to last weeks after cessation of antibiotic exposure.^31^ Second, as for many shotgun metagenomic sequencing analyses, the sequencing depth of our samples likely affected our ability to detect all ARGs present within a sample. To account for this, we included sequencing depth in all models for our primary analyses. Analyses of associations between individual antibiotic exposures and ARGs for specific antibiotic classes were not adjusted for sequencing depth or time relative to HCT because of model nonconvergence. Thus, while TMP-SMX exposure appears to reduce the richness of ARGs, this may reflect the fact that TMP-SMX was primarily administered prior to HCT and before the administration of broad-spectrum antibiotics for febrile neutropenia. Finally, with short-read sequencing data, we were unable to assign ARGs to specific bacterial species. Therefore, we can only make associations between gut microbiome composition and the gut resistome and cannot prove a causal link. Further work is needed to determine the effect of antibiotic exposures on ARGs assigned to specific bacterial species within the gut metagenome.

In conclusion, this study represents a large, longitudinal evaluation of the pediatric gut resistome among a severely immunocompromised population. Understanding the complex dynamics of the gut resistome during HCT and the effect of antibiotic exposures on the gut resistome can impact clinical decisions for antibiotic prophylaxis and treatment for children after HCT and other patient populations. Finally, this study provides further evidence of the importance of antimicrobial stewardship efforts for the prevention of promoting antibiotic resistance among hospitalized patients.

## Methods

### Study design and population

We conducted a prospective cohort study of children and adolescents (<18 years of age) who underwent their first HCT through the Duke University Pediatric Transplant and Cellular Therapy Program between October 2015 and February 2018. Subjects were prospectively enrolled during the pretransplantation evaluation, and fecal samples were collected from subjects on as many days as possible from 30 days before to 100 days after HCT. Daily clinical data, including information on receipt of antibiotics, were collected from the electronic medical record. Informed consent was obtained from participants’ legal guardians prior to enrollment, and the study protocol was approved by the Duke University Health System Institutional Review Board (Pro00064365).

### Transplant practices

Most children received TMP-SMX for *Pneumocystis jirovecii* prophylaxis from the start of the preparatory chemotherapy conditioning regimen through two days before HCT. Thereafter, children received monthly inhaled or IV pentamidine starting 30 days after HCT. After HCT, broad-spectrum antibiotics were initiated at onset of fever or concern for infection, but routine antibacterial prophylaxis was not provided. Cefepime was the first-line antibiotic for subjects experiencing fever and neutropenia; vancomycin or antibiotics with an anaerobic spectrum of activity (e.g., piperacillin-tazobactam, carbapenems) were also frequently used if there was concern for a specific infectious source or history of colonization or infection by antibiotic-resistant bacteria. Antibiotic selection and duration were at the discretion of the clinical provider.

### Metagenomic sequencing and data processing

We selected approximately weekly fecal samples from each subject for shotgun metagenomic sequencing. DNA was extracted using PowerSoil Pro Kits (Qiagen, Germantown, MD). DNA sequencing libraries were constructed using Nextera XT DNA Library Prep Kits (Illumina, San Diego, CA) and sequenced on NextSeq500 or NovaSeq6000 instruments (Illumina) as 150-bp paired-end reads. We trimmed reads using Trimmomatic (version 0.39) and removed host decontamination using Bowtie2 (version 2.3.5).^32,33^ Host-decontaminated reads were profiled for bacterial species relative abundances using MetaPhlAn 4.^34^ Using the ShortBRED pipeline (version 0.9.4), we aligned sequencing reads to the Comprehensive Antibiotic Resistance Database (CARD; version 3.2.7).^35,36^ Reads mapped to ARGs were normalized by dividing the proportion of sequencing reads mapped to a gene sequence by the length of the gene sequence to determine the reads per kilobase per million (RPKM). We pruned samples with less than 500,000 quality-filtered metagenomic read-pairs.

### Antibiotic exposures

We considered piperacillin-tazobactam, carbapenems, metronidazole, and clindamycin to have spectra of activity that included anaerobes. We considered antibiotics without appreciable anaerobic activity as aerobic antibiotics, specifically, cefepime, vancomycin, fluoroquinolones, aminoglycosides, macrolides, and TMP-SMX.^9,37^ A participant was considered exposed to an antibiotic if they received at least one dose of the antibiotic since the last sequenced fecal sample. If a participant received at least one dose of both an aerobic and an anaerobic antibiotic, they were considered to be exposed to both antibiotic exposure types. If a subject started antibiotics on the day of fecal sample collection, the antibiotic exposure was assigned to the next sequenced sample. Antibiotic exposures were included in statistical models as time-varying covariates with each interval representing time since the previously sequenced fecal sample.

### Statistical analyses

We used MaAsLin2 to fit linear mixed effects models to evaluate associations between antibiotic exposures, patient characteristics, transplant factors, and the relative abundances of specific bacterial species.^38^ For these adjusted models, we included subject as a random effect and limited analyses to species that had a minimum mean relative abundance of 1% and a sample prevalence of 5%; the false discovery rate was set to 0.1.

We assessed for the longitudinal effects of time during HCT and antibiotic exposures on gut microbiome composition and the gut resistome using adjusted mixed effects regression to model the number of species and ARGs, the number of new species and ARGs acquired since the previous sample, microbiome and resistome fluctuation as measured by the Jaccard distance, and the abundances of specific bacterial species and ARGs. An example of these regression formulas was Number of bacterial species ~ antibiotic exposure + age at HCT + sex + underlying diagnosis + preparatory conditioning regimen + type of HCT (autologous or allogeneic) + day relative to HCT + log of the sample sequencing depth + (1 | study subject). The Jaccard distance is a measure of dissimilarity between two samples, with values closer to one representing increased fluctuation.^39^ Models for the number of species and ARGs and the number of newly acquired species and ARGs were fit using a negative binomial distribution with the *glmmADMB* R package (version 0.8.3.3). The *lme4* R package (version 1.1–27.1) was used to model both linear regression for the Jaccard distance and gamma regression using a pseudocount and log link for the ARG abundance.^40,41^ We performed similar analyses to evaluate associations between exposures to specific antibiotics and measures of the resistome using adjusted mixed effects models. An example of these models was Number of ARGs ~ cefepime exposure + age at HCT + sex + underlying diagnosis + preparatory conditioning regimen + type of HCT (autologous or allogeneic) + day relative to HCT + log of the sample sequencing depth + (1 | study subject). Our findings were similar in sensitivity analyses that separately evaluated associations between the duration of antibiotic exposures and measures of the gut resistome and microbiome and that were limited to only allogeneic HCT recipients.

We then correlated measures of the resistome with corresponding measures of microbiome composition using repeated measures correlations implemented using the *rmcorr* R package (version 0.4.4).^42^ Finally, we used unadjusted mixed effect models to determine the effect of individual antibiotics on the number (negative binomial) and abundance (gamma regression) of ARGs encoding resistance to specific classes of antibiotics, with individual antibiotic exposures as fixed effects and subject as a random effect. An example of these models was Number of beta-lactam ARGs ~ cefepime exposure + (1 | study subject). We adjusted the false discovery rate using the Benjamini-Hochberg correction.^43^ All analyses were conducted using R statistical software (version 4.0.2).

## Supplementary Material

Supplemental Material

## Data Availability

The metagenomic data will be uploaded to the Sequence Read Archive prior to publication. The deidentified metadata and analytic script are also publicly available (https://github.com/smh114/Pediatric_HCT_Gut_Resistome).
