# Supplementary material for: The effects of antibiotic exposures on the gut resistome during hematopoietic cell transplantation in children
Source: Gut Microbes. 2024 Mar 30;16(1):2333748. doi: 10.1080/19490976.2024.2333748 (PMC10984140; doi:10.1080/19490976.2024.2333748)
Supplement: Supplemental Material [file KGMI_A_2333748_SM6975.docx]

| **Supplementary Table 1**. Significant associations of patient characteristics and antibiotic exposures and the relative abundances of bacterial species within the gut microbiome | | | | | | | |
| --- | --- | --- | --- | --- | --- | --- | --- |
| **Covariate** | | | **Species** | **Effect** | **Standard Error** | **P Value** | **Q Value** |
| **Antibiotic Exposures** | | |  |  |  |  |  |
|  | Cefepime | | *Bifidobacterium longum* | -2.1924 | 0.5248 | 0.0000 | 0.0011 |
|  | Cefepime | | *Blautia wexlerae* | -2.0208 | 0.3899 | 0.0000 | 0.0000 |
|  | Cefepime | | *Clostridia bacterium* | -1.7727 | 0.4531 | 1.00E-04 | 0.0029 |
|  | Cefepime | | *Erysipelatoclostridium ramosum* | -1.8731 | 0.5335 | 5.00E-04 | 0.0078 |
|  | Cefepime | | *Escherichia coli* | -1.1698 | 0.4454 | 0.0088 | 0.0762 |
|  | Cefepime | | *Eubacterium rectale* | -1.331 | 0.3102 | 0.0000 | 8.00E-04 |
|  | Cefepime | | *Faecalibacterium prausnitzii* | -1.9447 | 0.5024 | 1.00E-04 | 0.0031 |
|  | Cefepime | | *Fusicatenibacter saccharivorans* | -1.1692 | 0.3165 | 2.00E-04 | 0.0049 |
|  | Cefepime | | *Intestinibacter bartlettii* | -1.8751 | 0.3941 | 0.0000 | 1.00E-04 |
|  | Cefepime | | *Klebsiella pneumoniae* | -1.2115 | 0.4368 | 0.0057 | 0.0566 |
|  | Cefepime | | *Lachnospiraceae bacterium* | -3.0311 | 0.5202 | 0.0000 | 0.0000 |
|  | Cefepime | | *Roseburia intestinalis* | -0.7988 | 0.3038 | 0.0088 | 0.0762 |
|  | Cefepime | | *Ruminococcus gnavus* | -2.6902 | 0.5466 | 0.0000 | 1.00E-04 |
|  | Cefepime | | *Ruminococcus torques* | -1.9873 | 0.4009 | 0.0000 | 1.00E-04 |
|  | Cefepime | | *Streptococcus parasanguinis* | -2.0095 | 0.4557 | 0.0000 | 5.00E-04 |
|  | Cefepime | | *Streptococcus salivarius* | -2.537 | 0.4593 | 0.0000 | 0.0000 |
|  | Cefepime | | *Veillonella dispar* | -1.6698 | 0.4416 | 2.00E-04 | 0.004 |
|  | Cefepime | | *Veillonella parvula* | -2.1374 | 0.5299 | 1.00E-04 | 0.0018 |
|  | Fluoroquinolone | | *Bacteroides xylanisolvens* | 1.7165 | 0.6394 | 0.0074 | 0.0677 |
|  | Fluoroquinolone | | *Eggerthella lenta* | -3.2536 | 1.0294 | 0.0016 | 0.0214 |
|  | Fluoroquinolone | | *Intestinibacter bartlettii* | -2.1899 | 0.8138 | 0.0074 | 0.0676 |
|  | Fluoroquinolone | | *Phocaeicola dorei* | 1.7245 | 0.5598 | 0.0022 | 0.0262 |
|  | Fluoroquinolone | | *Phocaeicola massiliensis* | 1.6759 | 0.5908 | 0.0047 | 0.0483 |
|  | Macrolide | | *Bifidobacterium breve* | 2.0773 | 0.7845 | 0.0083 | 0.0742 |
|  | Metronidazole | | *Bacteroides fragilis* | -1.332 | 0.5216 | 0.0109 | 0.0914 |
|  | Metronidazole | | *Bacteroides uniformis* | -2.291 | 0.5976 | 1.00E-04 | 0.0034 |
|  | Metronidazole | | *Bilophila wadsworthia* | -0.8578 | 0.3136 | 0.0064 | 0.0614 |
|  | Metronidazole | | *Clostridioides difficile* | -1.6368 | 0.5158 | 0.0016 | 0.0208 |
|  | Metronidazole | | *Clostridium symbiosum* | -1.6492 | 0.5029 | 0.0011 | 0.0154 |
|  | Metronidazole | | *Enterocloster aldensis* | -1.7815 | 0.62 | 0.0042 | 0.0449 |
|  | Metronidazole | | *Enterocloster bolteae* | -1.9839 | 0.7517 | 0.0085 | 0.0752 |
|  | Metronidazole | | *Enterococcus casseliflavus* | 1.657 | 0.4401 | 2.00E-04 | 0.0041 |
|  | Metronidazole | | *Enterococcus faecalis* | 2.3425 | 0.6566 | 4.00E-04 | 0.0068 |
|  | Metronidazole | | *Enterococcus gallinarum* | 1.525 | 0.4487 | 7.00E-04 | 0.011 |
|  | Metronidazole | | *Flavonifractor plautii* | -2.5368 | 0.6536 | 1.00E-04 | 0.003 |
|  | Metronidazole | | *Lacticaseibacillus rhamnosus* | 1.4541 | 0.5234 | 0.0056 | 0.0563 |
|  | Metronidazole | | *Parabacteroides distasonis* | -1.7165 | 0.5676 | 0.0026 | 0.0302 |
|  | Metronidazole | | *Parabacteroides merdae* | -1.0239 | 0.3747 | 0.0065 | 0.0614 |
|  | Metronidazole | | *Phocaeicola vulgatus* | -2.0411 | 0.5654 | 3.00E-04 | 0.0064 |
|  | Other Antibiotics | | *Bacteroides xylanisolvens* | 1.512 | 0.3884 | 1.00E-04 | 0.003 |
|  | Other Antibiotics | | *Ruminococcus torques* | 1.5944 | 0.5378 | 0.0031 | 0.035 |
|  | Other Antibiotics | | *Streptococcus thermophilus* | 1.2534 | 0.4807 | 0.0094 | 0.0798 |
|  | Piperacillin-Tazobactam | | *Alistipes onderdonkii* | -2.9331 | 0.7112 | 0.0000 | 0.0013 |
|  | Piperacillin-Tazobactam | | *Bacteroides thetaiotaomicron* | -2.9358 | 0.6379 | 0.0000 | 3.00E-04 |
|  | Piperacillin-Tazobactam | | *Bacteroides xylanisolvens* | -1.2317 | 0.4222 | 0.0037 | 0.04 |
|  | Piperacillin-Tazobactam | | *Bifidobacterium longum* | -2.116 | 0.7701 | 0.0062 | 0.0601 |
|  | Piperacillin-Tazobactam | | *Bilophila wadsworthia* | -1.6224 | 0.3868 | 0.0000 | 0.0011 |
|  | Piperacillin-Tazobactam | | *Blautia wexlerae* | -1.496 | 0.57 | 0.0089 | 0.0762 |
|  | Piperacillin-Tazobactam | | *Clostridioides difficile* | -1.934 | 0.6321 | 0.0023 | 0.0277 |
|  | Piperacillin-Tazobactam | | *Dysosmobacter welbionis* | -1.6839 | 0.5529 | 0.0024 | 0.0285 |
|  | Piperacillin-Tazobactam | | *Enterococcus faecium* | 2.5532 | 0.7883 | 0.0013 | 0.0173 |
|  | Piperacillin-Tazobactam | | *Escherichia coli* | -2.2652 | 0.658 | 6.00E-04 | 0.0098 |
|  | Piperacillin-Tazobactam | | *Flavonifractor plautii* | -2.8483 | 0.7998 | 4.00E-04 | 0.0068 |
|  | Piperacillin-Tazobactam | | *Parabacteroides distasonis* | -2.6327 | 0.6996 | 2.00E-04 | 0.0041 |
|  | Piperacillin-Tazobactam | | *Parabacteroides merdae* | -1.452 | 0.4623 | 0.0018 | 0.0222 |
|  | Piperacillin-Tazobactam | | *Phocaeicola dorei* | -1.9531 | 0.3673 | 0.0000 | 0.0000 |
|  | Piperacillin-Tazobactam | | *Sutterella wadsworthensis* | -1.3993 | 0.3903 | 4.00E-04 | 0.0065 |
|  | Trimethoprim-Sulfamethoxazole | | *Bacteroides thetaiotaomicron* | -1.3168 | 0.4463 | 0.0033 | 0.0363 |
|  | Trimethoprim-Sulfamethoxazole | | *Bifidobacterium breve* | -1.1314 | 0.4391 | 0.0102 | 0.0863 |
|  | Trimethoprim-Sulfamethoxazole | | *Roseburia intestinalis* | 1.0835 | 0.3171 | 7.00E-04 | 0.0105 |
|  | Vancomycin | | *Clostridium symbiosum* | -1.2318 | 0.4488 | 0.0062 | 0.0602 |
|  | Vancomycin | | *Hungatella hathewayi* | -1.9563 | 0.6063 | 0.0013 | 0.0178 |
|  | Vancomycin | | *Phocaeicola massiliensis* | -0.6993 | 0.2765 | 0.0117 | 0.0975 |
|  | Vancomycin | | *Ruminococcus gnavus* | -1.6524 | 0.5856 | 0.0049 | 0.0498 |
| **Patient and Transplant Factors** | | |  |  |  |  |  |
|  | Age | | *Alistipes putredinis* | 1.1835 | 0.3962 | 0.0038 | 0.0415 |
|  | Age | | *Fusicatenibacter saccharivorans* | 0.7747 | 0.2122 | 5.00E-04 | 0.0079 |
|  | Age | | *Lacticaseibacillus rhamnosus* | 1.343 | 0.3887 | 9.00E-04 | 0.0137 |
|  | Age | | *Ruminococcaceae bacterium* | 1.2015 | 0.3431 | 8.00E-04 | 0.0121 |
|  | Allogeneic | | *Enterococcus casseliflavus* | -5.1395 | 1.6686 | 0.0028 | 0.032 |
|  | Allogeneic | | *Streptococcus parasanguinis* | -6.2819 | 2.0601 | 0.003 | 0.0341 |
|  | Allogeneic | | *Streptococcus thermophilus* | -5.1239 | 1.379 | 3.00E-04 | 0.0062 |
|  | **Diagnosis** | |  |  |  |  |  |
|  |  | Hematologic Non-Malignant Disease | *Alistipes onderdonkii* | 4.7318 | 1.4522 | 0.0017 | 0.0217 |
|  |  | Immunodeficiency | *Alistipes putredinis* | 3.5216 | 1.2688 | 0.0071 | 0.0664 |
|  |  | Immunodeficiency | *Clostridioides difficile* | -3.6509 | 1.2421 | 0.0046 | 0.0483 |
|  |  | Immunodeficiency | *Phocaeicola massiliensis* | 4.0697 | 1.2599 | 0.0019 | 0.0235 |
|  |  | Metabolic Disease | *Alistipes onderdonkii* | 4.6672 | 1.4283 | 0.0017 | 0.0214 |
|  |  | Metabolic Disease | *Alistipes putredinis* | 3.6462 | 0.9692 | 3.00E-04 | 0.0065 |
|  |  | Metabolic Disease | *Bacteroides uniformis* | 4.7523 | 1.2968 | 5.00E-04 | 0.0078 |
|  |  | Metabolic Disease | *Fusicatenibacter saccharivorans* | 1.5012 | 0.5146 | 0.0047 | 0.0483 |
|  |  | Metabolic Disease | GGB3293 SGB4348 | 1.8824 | 0.6868 | 0.0076 | 0.0687 |
|  |  | Metabolic Disease | *Phocaeicola massiliensis* | 3.829 | 0.9611 | 2.00E-04 | 0.0039 |
|  |  | Metabolic Disease | *Ruminococcaceae bacterium* | 2.3121 | 0.8373 | 0.0074 | 0.0676 |
|  |  | Solid Tumor | *Enterococcus casseliflavus* | -4.957 | 1.8354 | 0.0085 | 0.0752 |
|  |  | Solid Tumor | *Streptococcus thermophilus* | -4.661 | 1.4972 | 0.0024 | 0.0285 |
|  | Day Relative to HCT | | *Bacteroides fragilis* | -0.742 | 0.2001 | 2.00E-04 | 0.0047 |
|  | Day Relative to HCT | | *Bacteroides ovatus* | -0.7134 | 0.2161 | 0.001 | 0.0145 |
|  | Day Relative to HCT | | *Bacteroides thetaiotaomicron* | -0.5989 | 0.1984 | 0.0026 | 0.0305 |
|  | Day Relative to HCT | | *Bacteroides xylanisolvens* | -0.5833 | 0.1314 | 0.0000 | 5.00E-04 |
|  | Day Relative to HCT | | *Bifidobacterium longum* | -0.8588 | 0.2418 | 4.00E-04 | 0.0069 |
|  | Day Relative to HCT | | *Bilophila wadsworthia* | -0.5108 | 0.1202 | 0.0000 | 9.00E-04 |
|  | Day Relative to HCT | | *Blautia producta* | 0.6185 | 0.2249 | 0.0061 | 0.0601 |
|  | Day Relative to HCT | | *Clostridia bacterium* | 0.6948 | 0.2087 | 9.00E-04 | 0.0137 |
|  | Day Relative to HCT | | *Enterocloster aldensis* | 0.6761 | 0.2383 | 0.0047 | 0.0483 |
|  | Day Relative to HCT | | *Enterococcus casseliflavus* | 0.6062 | 0.1694 | 4.00E-04 | 0.0066 |
|  | Day Relative to HCT | | *Enterococcus faecalis* | 1.1103 | 0.2522 | 0.0000 | 5.00E-04 |
|  | Day Relative to HCT | | *Faecalibacterium prausnitzii* | -0.9027 | 0.2317 | 1.00E-04 | 0.003 |
|  | Day Relative to HCT | | *Intestinibacter bartlettii* | 1.0086 | 0.1818 | 0.0000 | 0.0000 |
|  | Day Relative to HCT | | *Klebsiella pneumoniae* | 0.6521 | 0.2014 | 0.0013 | 0.0173 |
|  | Day Relative to HCT | | *Lachnospiraceae bacterium* | 0.8618 | 0.2399 | 4.00E-04 | 0.0065 |
|  | Day Relative to HCT | | *Lacticaseibacillus rhamnosus* | 0.6844 | 0.2012 | 7.00E-04 | 0.011 |
|  | Day Relative to HCT | | *Parabacteroides distasonis* | -0.9955 | 0.2176 | 0.0000 | 3.00E-04 |
|  | Day Relative to HCT | | *Phocaeicola dorei* | -0.3552 | 0.114 | 0.0019 | 0.0236 |
|  | Day Relative to HCT | | *Phocaeicola vulgatus* | -0.7203 | 0.2169 | 9.00E-04 | 0.0137 |
|  | Day Relative to HCT | | *Ruminococcaceae bacterium* | 0.6888 | 0.1512 | 0.0000 | 3.00E-04 |
|  | Day Relative to HCT | | *Ruminococcus torques* | -0.6782 | 0.1848 | 3.00E-04 | 0.0052 |
|  | Day Relative to HCT | | *Streptococcus parasanguinis* | 1.905 | 0.2101 | 0.0000 | 0.0000 |
|  | Day Relative to HCT | | *Streptococcus salivarius* | 1.3733 | 0.2117 | 0.0000 | 0.0000 |
|  | Day Relative to HCT | | *Streptococcus thermophilus* | 0.6288 | 0.1696 | 2.00E-04 | 0.0047 |
|  | Day Relative to HCT | | *Sutterella wadsworthensis* | -0.3272 | 0.1213 | 0.0072 | 0.0668 |
|  | Day Relative to HCT | | *Veillonella dispar* | 1.2324 | 0.2036 | 0.0000 | 0.0000 |
|  | Day Relative to HCT | | *Veillonella parvula* | 1.271 | 0.2445 | 0.0000 | 0.0000 |
|  | Sequencing Depth | | *Bifidobacterium breve* | 0.56 | 0.175 | 0.0014 | 0.0192 |
|  | Sequencing Depth | | *Blautia producta* | 0.8491 | 0.2022 | 0.0000 | 0.0011 |
|  | Sequencing Depth | | *Clostridium innocuum* | 0.757 | 0.2109 | 4.00E-04 | 0.0065 |
|  | Sequencing Depth | | *Eggerthella lenta* | 0.8687 | 0.1953 | 0.0000 | 5.00E-04 |
|  | Sequencing Depth | | *Enterocloster clostridioformis* | 0.4986 | 0.1753 | 0.0046 | 0.0483 |
|  | Sequencing Depth | | *Enterococcus casseliflavus* | 0.8327 | 0.1525 | 0.0000 | 0.0000 |
|  | Sequencing Depth | | *Enterococcus faecalis* | 1.4247 | 0.2264 | 0.0000 | 0.0000 |
|  | Sequencing Depth | | *Enterococcus faecium* | 1.097 | 0.2216 | 0.0000 | 1.00E-04 |
|  | Sequencing Depth | | *Enterococcus gallinarum* | 0.7737 | 0.1544 | 0.0000 | 1.00E-04 |
|  | Sequencing Depth | | *Intestinibacter bartlettii* | 0.6138 | 0.1644 | 2.00E-04 | 0.0045 |
|  | Sequencing Depth | | *Klebsiella pneumoniae* | 0.882 | 0.1814 | 0.0000 | 1.00E-04 |
|  | Sequencing Depth | | *Lachnospiraceae bacterium* | 0.831 | 0.2161 | 1.00E-04 | 0.0033 |
|  | Sequencing Depth | | *Lacticaseibacillus rhamnosus* | 0.9343 | 0.1808 | 0.0000 | 0.0000 |
|  | Sequencing Depth | | *Sellimonas intestinalis* | 0.7527 | 0.2008 | 2.00E-04 | 0.0043 |
|  | Sequencing Depth | | *Streptococcus parasanguinis* | 0.8123 | 0.1892 | 0.0000 | 8.00E-04 |
|  | Sequencing Depth | | *Streptococcus salivarius* | 0.7703 | 0.1905 | 1.00E-04 | 0.0018 |
|  | Sequencing Depth | | *Streptococcus thermophilus* | 0.9261 | 0.1536 | 0.0000 | 0.0000 |
|  | Sequencing Depth | | *Veillonella dispar* | 0.7911 | 0.1832 | 0.0000 | 7.00E-04 |
|  | Sequencing Depth | | *Veillonella parvula* | 0.9027 | 0.2212 | 1.00E-04 | 0.0016 |
| HCT; hematopoietic cell transplantation; Other antibiotics includes exposures to tetracyclines, clindamycin, aminoglycosides, and non-cefepime beta-lactams. | | | | | | | |

| **Supplementary Table 2.** Effect of clinical factors on measures of the resistome and microbiome | | | | | | | | |
| --- | --- | --- | --- | --- | --- | --- | --- | --- |
|  | | **Resistome**  β (95% CI)  P value | | | | **Microbiome**  β (95% CI)  P value | | |
| **Clinical Factors** | | **ARG Abundance** | **Number of ARGs** | **Number of New ARGs** | **Jaccard Distance ARGs** | **Number of Species** | **Number of New Species** | **Jaccard Distance Species** |
| Antibiotic Exposures | |  |  |  |  |  |  |  |
|  | None | Reference | | | | Reference | | |
|  | Aerobic | 1.08 (0.90, 1.30)  P=0.40 | 0.81 (0.74, 0.90)  P<0.0001 | 1.09 (0.91, 1.30)  P=0.33 | 0.02 (-0.03, 0.08)  P=0.41 | 0.71 (0.64, 0.79)  P<0.0001 | 1.05 (0.89, 1.25)  P=0.54 | 0.10 (0.05, 0.14)  P<0.0001 |
|  | Anaerobic | 1.62 (1.15, 2.27)  P=0.005 | 0.73 (0.61, 0.88)  P=0.0008 | 1.50 (1.12, 2.01)  P=0.006 | 0.13 (0.04, 0.22)  P=0.003 | 0.66 (0.53, 0.82)  P=0.0001 | 1.45 (1.10, 1.90)  P=0.008 | 0.15 (0.07, 0.22)  P=0.0001 |
|  | Both | 2.16 (1.73, 2.68)  P<0.001 | 0.70 (0.62, 0.80)  P<0.0001 | 1.26 (1.02, 1.55)  P=0.03 | 0.08 (0.02, 0.14)  P=0.008 | 0.46 (0.40, 0.54)  P<0.0001 | 1.08 (0.88, 1.32)  P=0.47 | 0.17 (0.12, 0.22)  P<0.0001 |
| Subject Age | | 0.95 (0.92, 0.99)  P=0.01 | 1.00 (0.98, 1.02)  P=0.80 | 0.99 (0.97, 1.00)  P=0.10 | -0.008 (-0.01, -0.001)  P=0.03 | 1.04 (1.02, 1.06)  P<0.0001 | - 1. (0.99, 1.03)   P=0.26 | -0.005 (-0.01, 0.00)  P=0.09 |
| Sex | |  |  |  |  |  |  |  |
|  | Female | Reference | | | | Reference | | |
|  | Male | 1.13 (0.76, 1.68)  P=0.55 | 1.06 (0.88, 1.27)  P=0.53 | 1.10 (0.94, 1.30)  P=0.24 | -0.008 (-0.08, 0.06)  P=0.82 | 0.95 (0.79, 1.15)  P=0.59 | 1.01 (0.86, 1.18)  P=0.94 | -0.02 (-0.07, 0.03)  P=0.46 |
| Diagnosis | |  |  |  |  |  |  |  |
|  | Hematologic Malignancy | Reference | | | | Reference | | |
|  | Congenital Immunodeficiency | 0.69 (0.34, 1.43)  P=0.32 | 1.19 (0.87, 1.65)  P=0.28 | 1.28 (0.97, 1.69)  P=0.09 | -0.02 (-0.14, 0.10)  P=0.78 | 1.60 (1.14, 2.24)  P=0.006 | 1.30 (0.98, 1.72)  P=0.07 | -0.06 (-0.16, 0.03)  P=0.20 |
|  | Non-malignant Heme | 1.18 (0.67, 2.07)  P=0.56 | 1.02 (0.79, 1.31)  P=0.89 | 0.95 (0.76, 1.19)  P=0.65 | -0.06 (-0.16, 0.03)  P=0.21 | 1.28 (0.99, 1.67)  P=0.06 | 0.98 (0.78, 1.22)  P=0.85 | -0.08 (-0.15, -0.006)  P=0.05 |
|  | Metabolic Disorder | 0.83 (0.48, 1.44)  P=0.50 | 0.96 (0.75, 1.24)  P=0.77 | 1.04 (0.83, 1.31)  P=0.73 | -0.03 (-0.12, 0.06)  P=0.54 | 1.57 (1.21, 2.04)  P=0.0008 | 1.16 (0.92, 1.46)  P=0.21 | -0.05 (-0.12, 0.02)  P=0.23 |
|  | Solid Tumor | 0.40 (0.10, 1.62)  P=0.20 | 0.92 (0.49, 1.72)  P=0.79 | 0.76 (0.41, 1.42)  P=0.39 | -0.03 (-0.28, 0.22)  P=0.84 | 1.36 (0.71, 2.62)  P=0.35 | 0.85 (0.45, 1.60)  P=0.61 | -0.03 (-0.22, 0.16)  P=0.74 |
| Preparatory Regimen | |  |  |  |  |  |  |  |
|  | Myeloablative | Reference | | | | Reference | | |
|  | Non-myeloablative/RIC | 0.86 (0.29, 2.55)  P=0.79 | 0.96 (0.58, 1.62)  P=0.89 | 0.87 (0.49, 1.55)  P=0.65 | -0.06 (-0.26, 0.14)  P=0.57 | 1.11 (0.65, 1.90)  P=0.70 | 0.87 (0.47, 1.52)  P=0.63 | -0.11 (-0.27, 0.04)  P=0.18 |
| Type of HCT | |  |  |  |  |  |  |  |
|  | Autologous | Reference | | | | Reference | | |
|  | Allogeneic | 0.57 (0.16, 1.98)  P=0.37 | 0.76 (0.43, 1.34)  P=0.34 | 0.78 (0.43, 1.42)  P=0.42 | 0.03 (-0.20, 0.25)  P=0.83 | 0.72 (0.40, 1.30)  P=0.27 | 0.85 (0.47, 1.54)  P=0.59 | 0.05 (-0.13, 0.22)  P=0.62 |
| Day Relative to HCT | | 1.004 (1.001, 1.007)  P=0.009 | 1.002 (1.001, 1.004)  P=0.003 | 1.008 (1.006, 1.011)  P<0.0001 | -0.001 (-0.002, -0.000)  P=0.04 | 1.000 (1.000, 1.002)  P=0.63 | 1.010 (1.008, 1.012)  P<0.0001 | -0.000 (-0.001, 0.000)  P=0.43 |
| Log of Sequencing Depth | | 1.01 (0.92, 1.10)  P=0.85 | 1.31 (1.25, 1.38)  P<0.0001 | 1.37 (1.25, 1.51)  P<0.0001 | -0.02 (-0.04, 0.003)  P=0.09 | 1.29 (1.22, 1.37)  P<0.0001 | 1.33 (1.22, 1.45)  P<0.0001 | -0.04 (-0.06, -0.02)  P<0.0001 |
| CI, confidence interval; ARG, antibiotic resistance gene; Non-malignant Heme, non-malignant hematologic disorder; RIC, reduced intensity conditioning; HCT, hematopoietic cell transplantation | | | | | | | | |

| **Supplementary Table 3.** Effect of antibiotics on the resistome | | | | |
| --- | --- | --- | --- | --- |
| **Antibiotic Exposure** | **Resistome**  β (95% CI)  P value | | | |
|  | **ARG Abundance** | **Number of ARGs** | **Number of New ARGs** | **Jaccard Distance ARGs** |
| Cefepime | 1.24 (1.06, 1.45)  P=0.008 | 0.79 (0.72, 0.86)  P<0.0001 | 1.34 (1.15, 1.55)  P=0.0001 | 0.06 (0.02, 0.10)  P=0.005 |
| Vancomycin | 1.57 (1.33, 1.86)  P<0.0001 | 0.83 (0.75, 0.91)  P=0.0001 | 1.25 (1.06, 1.46)  P=0.01 | 0.06 (0.02, 0.10)  P=0.01 |
| Fluoroquinolone | 1.30 (0.87, 1.96)  P=0.20 | 1. 01 (0.88, 1.37)  P=0.41 | 0.83 (0.58, 1.18)  P=0.29 | -0.10 (-0.21, 0.00)  P=0.06 |
| Aminoglycoside | 0.84 (0.50, 1.41)  P=0.51 | 0.87 (0.62, 1.22)  P=0.42 | 1.01 (0.60, 1.71)  P=0.96 | 0.13 (-0.02, 0.26)  P=0.08 |
| Macrolide | 1.09 (0.76, 1.56)  P=0.64 | 1.02 (0.84, 1.24)  P=0.83 | 1.14 (0.85, 1.53)  P=0.39 | 0.05 (-0.04, 0.14)  P=0.31 |
| TMP-SMX | 0.90 (0.74, 1.11)  P=0.32 | 1.03 (0.92, 1.16)  P=0.58 | 0.80 (0.65, 0.99)  P=0.04 | 0.02 (-0.04, 0.07)  P=0.58 |
| Pip-Tazo | 2.08 (1.55, 2.79)  P<0.0001 | 0.75 (0.65, 0.88)  P=0.0004 | 1.42 (1.12, 1.80)  P=0.003 | 0.12 (0.05, 0.19)  P=0.0007 |
| Carbapenem | 1.41 (0.92, 2.15)  P=0.12 | 1.02 (0.79, 1.33)  P=0.87 | 1.00 (0.66, 1.51)  P=0.99 | 0.04 (-0.07, 0.15)  P=0.44 |
| Metronidazole | 1.91 (1.53, 2.38)  P<0.0001 | 0.84 (0.74, 0.96)  P=0.01 | 1.29 (1.07, 1.56)  P=0.01 | 0.09 (0.03, 0.15)  P=0.002 |
| Clindamycin | 1.30 (0.69, 2.47)  P=0.42 | 0.81 (0.55, 1.19)  P=0.29 | 1.00 (0.56, 1.75)  P=0.99 | 0.01 (-0.15, 0.17)  P=0.89 |
| CI, confidence interval; ARG, antibiotic resistance gene; TMP-SMX, trimethoprim-sulfamethoxazole; Pip-Tazo, piperacillin-tazobactam | | | | |

| **Supplementary Table 4.** Effect of antibiotic exposure on the number of antibiotic resistance genes by antibiotic class | | | | | | | | | | |
| --- | --- | --- | --- | --- | --- | --- | --- | --- | --- | --- |
| **Antibiotic Exposure** | **Antibiotic Class**  β (95% CI)  Adjusted P value | | | | | | | | | |
|  | **Beta-Lactam** | **Carbapenem** | **Glycopeptide** | **Nitroimidazole** | **Fluoroquinolone** | **Diaminopyrimidine** | **Macrolide** | **Tetracycline** | **Aminoglycoside** | **Lincosamide** |
| Cefepime | 0.66 (0.57, 0.76)  P<0.0001 | 0.68 (0.58, 0.80)  P<0.0001 | 0.99 (0.80, 1.23)  P=0.93 | 0.58 (0.43, 0.77)  P=0.0005 | 0.69 (0.60, 0.80)  P<0.0001 | 0.86 (0.77, 0.98)  P=0.03 | 0.81 (0.73, 0.89)  P<0.0001 | 0.69 (0.62, 0.76)  P<0.0001 | 0.74 (0.65, 0.84)  P<0.0001 | 0.78 (0.70, 0.86)  P<0.0001 |
| Pip-Tazo | 0.56 (0.44, 0.72) P<0.0001 | 0.74 (0.56, 0.99)  P=0.06 | 1.32 (0.92, 1.89)  P=0.15 | 0.70 (0.44, 1.11)  P=0.15 | 0.70 (0.55, 0.89)  P=0.008 | 1.00 (0.81, 1.23)  P=0.99 | 0.77 (0.65, 0.92)  P=0.008 | 0.65 (0.54, 0.77)  P<0.0001 | 0.78 (0.63, 0.95)  P=0.03 | 0.75 (0.62, 0.92)  P=0.008 |
| Carbapenem | 1.04 (0.70, 1.55)  P=0.90 | 1.20 (0.80, 1.80)  P=0.60 | 1.75 (1.01, 3.04)  P=0.23 | 0.64 (0.27, 1.52)  P=0.60 | 1.07 (0.72, 1.58)  P=0.84 | 1.38 (1.03, 1.85)  P=0.23 | 1.10 (0.82, 1.46) P=0.67 | 0.80 (0.60, 1.08)  P=0.41 | 1.17 (0.82, 1.65)  P=0.60 | 0.99 (0.73, 1.36)  P=0.97 |
| Vancomycin | 0.72 (0.62, 0.84)  P=0.0001 | 0.80 (0.67, 0.95)  P=0.02 | 0.98 (0.78, 1.22)  P=0.87 | 0.60 (0.43, 0.83)  P=0.004 | 0.75 (0.64, 0.88)  P=0.0008 | 0.90 (0.79, 1.02)  P=0.12 | 0.82 (0.73, 0.91)  P=0.0008 | 0.71 (0.64, 0.79)  P<0.0001 | 0.79 (0.69, 0.91)  P=0.001 | 0.74 (0.66, 0.84)  P<0.0001 |
| Metronidazole | 0.64 (0.52, 0.80)  P=0.0003 | 0.79 (0.62, 1.00)  P=0.06 | 1.35 (1.01, 1.80)  P=0.06 | 0.76 (0.51, 1.14)  P=0.19 | 0.87 (0.71, 1.07)  P=0.19 | 0.99 (0.83, 1.18)  P=0.89 | 0.83 (0.71, 0.96)  P=0.02 | 0.69 (0.59, 0.80)  P<0.0001 | 0.83 (0.70, 0.99)  P=0.06 | 0.75 (0.64, 0.88)  P=0.0008 |
| Fluoroquinolone | 1.31 (0.94, 1.84)  P=0.33 | 1.32 (0.90, 1.93)  P=0.33 | 0.95 (0.44, 2.07)  P=0.91 | 0.95 (0.48, 1.90)  P=0.91 | 1.27 (0.87, 1.84)  P=0.43 | 1.15 (0.82, 1.62)  P=0.56 | 1.22 (0.94, 1.58)  P=0.33 | 1.25 (0.99, 1.59)  P=0.32 | 1.26 (0.93, 1.72)  P=0.33 | 1.11 (0.84, 1.45)  P=0.58 |
| TMP-SMX | 0.93 (0.79, 1.09)  P=0.42 | 0.81 (0.67, 0.97)  P=0.04 | 0.59 (0.45, 0.77)  P=0.0005 | 0.88 (0.64, 1.20)  P=0.44 | 0.73 (0.62, 0.87)  P=0.002 | 0.89 (0.77, 1.03)  P=0.15 | 0.83 (0.74, 0.94)  P=0.007 | 0.90 (0.80, 1.00)  P=0.08 | 0.85 (0.74, 0.98)  P=0.04 | 0.79 (0.70, 0.89)  P=0.0007 |
| Macrolide | 1.10 (0.79, 1.52)  P=0.88 | 1.02 (0.71, 1.48)  P=1.00 | 1.60 (1.05, 2.46)  P=0.20 | 1.00 (0.55, 1.82)  P=1.00 | 1.07 (0.77, 1.49)  P=0.88 | 1.13 (0.86, 1.47)  P=0.88 | 1.09 (0.86, 1.37)  P=0.26 | 1.04 (0.83, 1.31)  P=0.88 | 1.15 (0.87, 1.51)  P=0.88 | 1.38 (1.12, 1.70)  P=0.02 |
| Other° | 1.06 (0.85, 1.32)  P=0.88 | 1.25 (0.98, 1.59)  P=0.33 | 1.08 (0.73, 1.60)  P=0.90 | 1.14 (0.75, 1.72)  P=0.88 | 1.03 (0.81, 1.31)  P=0.90 | 1.06 (0.86, 1.31)  P=0.88 | 1.00 (0.84, 1.18)  P=0.97 | 0.98 (0.83, 1.15)  P=0.90 | 1.14 (0.93, 1.39)  P=0.60 | 0.92 (0.77, 1.10)  P=0.83 |
| °Other includes clindamycin, tetracycline, non-cefepime beta-lactams, and aminoglycoside exposures.  ARG, antibiotic resistance gene; CI, confidence interval; Pip-Tazo, piperacillin-tazobactam; TMP-SMX, trimethoprim-sulfamethoxazole | | | | | | | | | | |

| **Supplementary Table 5.** Effect of antibiotic exposure on the abundance of antibiotic resistance genes by antibiotic class | | | | | | | | | | |
| --- | --- | --- | --- | --- | --- | --- | --- | --- | --- | --- |
| **Antibiotic Exposure** | **Antibiotic Class**  β (95% CI)  Adjusted P value | | | | | | | | | |
|  | **Beta-Lactam** | **Carbapenem** | **Glycopeptide** | **Nitroimidazole** | **Fluoroquinolone** | **Diaminopyrimidine** | **Macrolide** | **Tetracycline** | **Aminoglycoside** | **Lincosamide** |
| Cefepime | 0.90 (0.69, 1.18)  P=0.49 | 0.83 (0.83, 0.84)  P<0.0001 | 1.34 (1.02, 1.76)  P=0.05 | 0.62 (0.47, 0.82)  P=0.002 | 1.15 (0.86, 1.54)  P=0.41 | 0.96 (0.76, 1.22)  P=0.79 | 1.30 (1.08, 1.58)  P=0.01 | 0.92 (0.78, 1.09)  P=0.41 | 1.17 (0.90, 1.52)  P=0.31 | 1.33 (1.10, 1.61)  P=0.005 |
| Pip-Tazo | 1.13 (0.68, 1.86)  P=0.67 | 1.83 (1.06, 3.17)  P=0.04 | 2.22 (1.29, 3.83)  P=0.008 | 0.38 (0.22, 0.66)  P=0.002 | 1.56 (0.94, 2.58)  P=0.11 | 2.37 (1.58, 3.55)  P=0.0001 | 1.77 (1.73, 1.81)  P<0.0001 | 2.11 (1.52, 2.93)  P<0.0001 | 1.98 (1.24, 3.16)  P=0.008 | 1.79 (1.29, 2.49) P=0.002 |
| Carbapenem | 1.53 (0.76, 3.09)  P=0.56 | 1.45 (0.66, 3.15)  P=0.60 | 2.10 (0.99, 4.43)  P=0.23 | 0.78 (0.34, 1.75)  P=0.67 | 1.83 (0.84, 3.99)  P=0.41 | 1.41 (0.78, 2.54)  P=0.56 | 1.78 (1.08, 2.93)  P=0.23 | 1.17 (0.73, 1.87)  P=0.67 | 1.16 (0.59, 2.28)  P=0.79 | 1.63 (0.99, 2.69)  P=0.23 |
| Vancomycin | 1.48 (1.46, 1.49)  P<0.0001 | 1.57 (1.15, 2.15)  P=0.008 | 1.33 (0.99, 1.77)  P=0.07 | 1.00 (0.74, 1.36)  P=0.99 | 1.58 (1.16, 2.15)  P=0.006 | 1.10 (0.85, 1.41)  P=0.53 | 1.49 (1.22, 1.81)  P=0.0003 | 1.41 (1.39, 1.43)  P<0.0001 | 1.58 (1.57, 1.58)  P<0.0001 | 1.24 (1.02, 1.52)  P=0.05 |
| Metronidazole | 1.63 (1.63, 1.64)  P<0.0001 | 2.43 (1.62, 3.66)  P<0.0001 | 2.10 (1.44, 3.07)  P=0.0003 | 1.56 (1.03, 2.37)  P=0.05 | 3.53 (2.34, 5.33)  P<0.0001 | 2.04 (1.48, 2.81)  P<0.0001 | 2.28 (1.75, 2.97)  P<0.0001 | 1.22 (0.96, 1.55)  P=0.12 | 2.18 (1.53, 3.11)  P<0.0001 | 1.66 (1.28, 2.15)  P=0.0003 |
| Fluoroquinolone | 1.24 (0.64, 2.37)  P=0.62 | 1.46 (0.70, 3.03)  P=0.51 | 0.73 (0.34, 1.56)  P=0.56 | 0.55 (0.27, 1.10)  P=0.33 | 1.57 (0.73, 3.37)  P=0.46 | 1.99 (1.04, 3.83)  P=0.26 | 1.91 (1.89, 1.94)  P<0.0001 | 1.22 (0.79, 1.89)  P=0.56 | 1.12 (0.59, 2.11)  P=0.81 | 1.84 (1.15, 2.95)  P=0.12 |
| TMP-SMX | 1.19 (0.85, 1.65)  P=0.39 | 0.84 (0.84, 0.85)  P<0.0001 | 0.66 (0.50, 0.88)  P=0.009 | 1.05 (0.73, 1.51)  P=0.81 | 0.60 (0.41, 0.87)  P=0.01 | 0.74 (0.74, 0.74)  P<0.0001 | 0.72 (0.57, 0.90)  P=0.009 | 0.92 (0.75, 1.12)  P=0.44 | 0.77 (0.57, 1.05)  P=0.14 | 0.76 (0.75, 0.76)  P<0.0001 |
| Macrolide | 0.99 (0.54, 1.81)  P=1.00 | 1.18 (0.59, 2.35)  P=0.88 | 4.06 (2.18, 7.55)  P=0.0002 | 1.26 (0.63, 2.52)  P=0.88 | 1.25 (0.66, 2.37)  P=0.88 | 1.14 (0.67, 1.94)  P=0.88 | 1.07 (0.70, 1.62)  P=0.90 | 1.09 (0.73, 1.62)  P=0.88 | 1.30 (0.73, 2.31)  P=0.88 | 1.28 (0.84, 1.94)  P=0.88 |
| Other° | 0.98 (0.63, 1.53)  P=0.97 | 1.18 (0.74, 1.89)  P=0.88 | 0.93 (0.60, 1.45)  P=0.90 | 1.51 (0.95, 2.41)  P=0.33 | 1.19 (0.73, 1.94)  P=0.88 | 1.34 (0.91, 1.99)  P=0.46 | 1.33 (0.97, 1.82)  P=0.33 | 1.17 (0.88, 1.55)  P=0.69 | 1.61 (1.60, 1.62)  P<0.0001 | 1.23 (1.22, 1.24)  P<0.0001 |
| °Other includes clindamycin, tetracycline, non-cefepime beta-lactams, and aminoglycoside exposures.  ARG, antibiotic resistance gene; CI, confidence interval; Pip-Tazo, piperacillin-tazobactam; TMP-SMX, trimethoprim-sulfamethoxazole | | | | | | | | | | |

**Supplementary Figure 1.**

**Changes to the relative abundance of ARGs over time by clinical outcome**. A. Relative abundance of ARGs from the gut resistome of participants who did and did not develop acute GVHD of the gut or liver; B. Relative abundance of ARGs from the gut resistome of participants who did and did not develop bloodstream infections; C. Relative abundance of ARGs from the gut resistome of participants who did and did not experience two-year all-cause mortality. There are no obvious differences in the abundance of ARGs between participants who experienced different clinical outcomes. Points represent individual fecal samples; the smoothed lines were created using the Loess function, and shaded areas represent the 95% confidence intervals. ARG, antibiotic resistance gene; HCT, hematopoietic cell transplantation.

**Supplementary Figure 1.**

**
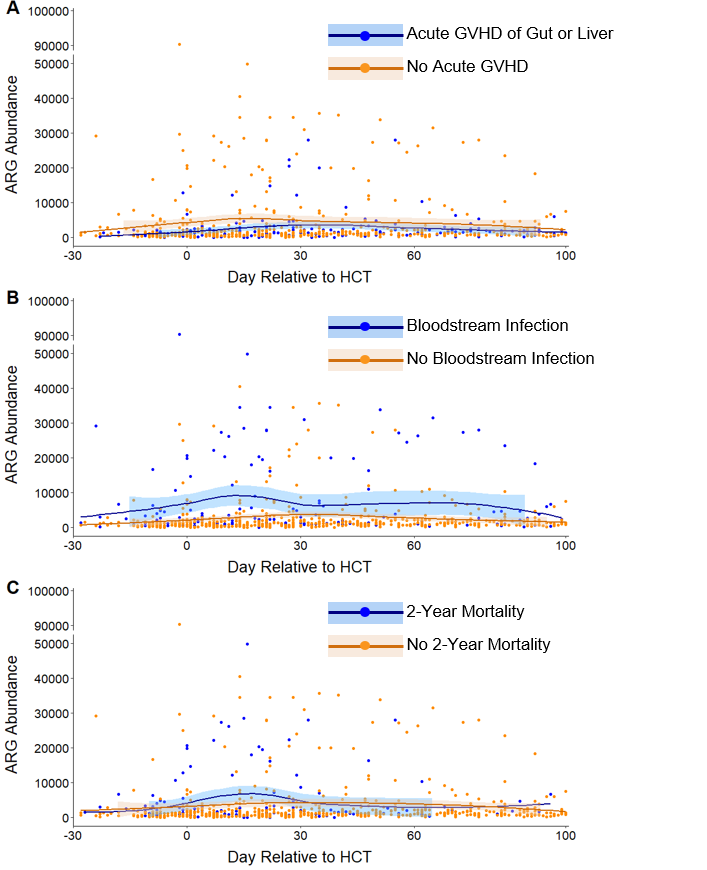
**
